# CP91110P: A Computationally Designed Multi-Epitope Vaccine Candidate for Tuberculosis via TLR-2/4 Synergistic Immunomodulation

**DOI:** 10.3390/biology14091196

**Published:** 2025-09-05

**Authors:** Yajing An, Syed Luqman Ali, Yanhua Liu, Aigul Abduldayeva, Ruizi Ni, Yufeng Li, Mingming Zhang, Yuan Tian, Lina Jiang, Wenping Gong

**Affiliations:** 1 Beijing Key Laboratory of New Techniques of Tuberculosis Diagnosis and Treatment, Institute of Tuberculosis, Senior Department of Tuberculosis, The Eighth Medical Center of PLA General Hospital, Beijing 100091, China; anyajing@asu.edu.pl (Y.A.); 1707025045@stu.sqxy.edu.cn (Y.L.); 2 Graduate School, Hebei North University, Zhangjiakou 075000, China; ruizini@asu.edu.pl (R.N.); liyufeng@stu.sqxy.edu.cn (Y.L.); zhangmingming@stu.sqxy.edu.cn (M.Z.); tianyuan@stu.sqxy.edu.cn (Y.T.); 3 Department of Biochemistry, Abdul Wali Khan University, Mardan 23200, Pakistan; luqmanali@awkum.edu.pk; 4 Research Institute of Preventive Medicine named Academician E. Dalenov, Astana Medical University, Astana 010000, Kazakhstan; abduldayeva.a@amu.kz; 5 Department of Immunology, College of Laboratory Medicine & Institute of Microcirculation, Hebei North University, Zhangjiakou 075000, China

**Keywords:** tuberculosis, multi-epitope vaccine, bioinformatics, immunoinformatics, immunogenicity

## Abstract

**Background**: Tuberculosis (TB) remains a global health priority, with current interventions like the Bacille Calmette–Guérin (BCG) vaccine lacking efficacy against latent infection and drug-resistant strains. Novel vaccines targeting both latent and active TB are urgently needed. **Objective**: This study aims to design a multi-epitope vaccine (MEV) and evaluate its immunogenicity, structural stability, and interactions with toll-like receptor 2/4 (TLR-2/4) via computational biology approaches. **Methods**: We designed MEV using bioinformatics tools, prioritizing immunodominant epitopes from *Mycobacterium tuberculosis* antigens. Structural stability was optimized through disulfide engineering, and molecular docking/dynamics simulations were used to analyze interactions and conformational dynamics with TLR-2/4. Antigenicity, immunogenicity, population coverage, and immune responses were computationally assessed. **Results**: The MEV candidate, CP91110P, exhibited 86.18% predicted global human leukocyte antigen (HLA)-I/II coverage, high antigenicity (VaxiJen: 0.8789), and immunogenicity (IEDB: 4.40091), with favorable stability (instability index: 33.48) and solubility (0.485). Tertiary structure analysis indicated that 98.34% residues were located in favored regions. Molecular docking suggested strong TLR-2 (−1535.9 kcal/mol) and TLR-4 (−1672.5 kcal/mol) binding. Molecular dynamics simulations indicated stable TLR-2 interactions (RMSD: 6–8 Å; Rg: 38.50–39.50 Å) and flexible TLR-4 binding (RMSD: 2–6 Å; Rg: 33–36 Å). Principal component analysis, free energy landscapes, and dynamic cross-correlation matrix analyses highlighted TLR-2’s structural coherence versus TLR-4’s adaptive flexibility. Immune simulations predicted potential robust natural killer cell activation, T helper 1 polarization (interferon-gamma/interleukin-2 dominance), and elevated IgM/IgG levels. **Conclusions**: CP91110P is predicted to stably bind to TLR-2 and flexibly interact with TLR-4, with prediction of its high antigenicity and broad coverage across immune populations. However, this conclusion requires confirmation through experimental validation. Therefore, it may provide a promising candidate for experimental validation in the development of tuberculosis vaccines.

## Simple Summary

Tuberculosis (TB) remains a major global health threat, causing over a million deaths yearly, especially in poorer regions. The current Bacille Calmette–Guérin (BCG) vaccine does not reliably prevent latent (hidden) infections or drug-resistant TB. To address this, we designed a new vaccine candidate called CP91110P using computer-based methods. This vaccine combines key pieces (epitopes) from both active and dormant TB bacteria to train the immune system. It also includes special boosters (adjuvants) to strengthen immune responses. Computer tests suggested that CP91110P may be safe and highly recognizable to the immune system (antigenic) and may work across diverse populations (covering ~86% of people worldwide). It exhibited strong binding potential to key immune receptors (TLR-2 and TLR-4), could potentially trigger protective immune cell activity (like Natural Killer cells and T-cells), and may promote helpful antibodies. Simulations also predicted that it would effectively fight TB inside cells. The computer simulation results suggest that this research may offer a promising new vaccine design that may prevent both active tuberculosis and latent tuberculosis. However, further laboratory and clinical tests are needed to confirm these results.

## 1. Introduction

Tuberculosis (TB) remains a significant global public health challenge, being one of the leading causes of morbidity and mortality from infectious diseases [1,2,3]. According to the World Health Organization (WHO), approximately 10.8 million cases and 1.25 million deaths attributed to TB were reported in 2023, with a substantial burden concentrated in low- and middle-income countries (LMICs) where socioeconomic disparities exacerbate the impact of the disease [4]. Despite the availability of the Bacillus Calmette–Guérin (BCG) vaccine and antibiotic therapies, the effectiveness of current TB control strategies remains insufficient [5,6,7]. The BCG vaccine exhibits variable efficacy, particularly against adult pulmonary TB, and fails to prevent the reactivation of latent TB infections, underscoring the imperative for improved preventive measures [8,9,10]. Furthermore, the emergence of multidrug-resistant (MDR) and extensively drug-resistant (XDR) TB strains complicates treatment regimens, posing significant challenges to global TB control efforts [11].

Current diagnostic methods for TB also present significant limitations, particularly in detecting latent infections and drug-resistant strains, which hamper timely intervention and management strategies [3,12,13]. These diagnostic gaps highlight the need for innovative approaches, including the development of novel vaccines that target both latent and active TB. Multi-epitope vaccines (MEVs) represent a promising solution, with the potential to elicit broad immune responses, enhance cost-effectiveness, and adapt to diverse *Mycobacterium tuberculosis* (MTB) strains [6,14,15,16,17,18,19,20,21]. The increasing recognition of the importance of immune evasion mechanisms employed by MTB further emphasizes the necessity for vaccines that can target multiple epitopes to provide comprehensive protection [22,23].

Advancements in bioinformatics and epitope prediction algorithms have emerged as pivotal tools to address the empirical limitations of traditional antigen screening methods, which are often time-consuming and costly [24,25]. By prioritizing immunodominant epitopes, researchers can enhance the likelihood of eliciting cross-reactive T and B cell immune responses [26,27]. However, previous studies have largely overlooked the integration of antigens associated with both latency and active infection phases, as well as the diversity of human leukocyte antigen (HLA) profiles critical for global vaccine applicability. Our proposed study aims to bridge these gaps by focusing on the prioritization of epitopes derived from both latent (e.g., Rv2031c, which is highly expressed under hypoxic conditions during latency with basal expression in active phases) and active-phase (e.g., Ag85A/B, predominantly upregulated during active bacterial proliferation) antigens, while also integrating toll-like receptor (TLR) agonists to foster a robust immune response that engages both innate and adaptive immune systems.

The primary objective of this research is to design a novel multi-epitope vaccine, designated CP91110P, that is predicted to exhibit favorable stability, immunogenicity, and global applicability. To achieve this, a multi-layered screening approach will be employed, combining assessments of major histocompatibility complex (MHC) binding, antigenicity, cytokine induction, and safety profiling. Structural optimization strategies, including disulfide engineering and molecular dynamics simulations, will be utilized to potentially ensure the stability of the vaccine construct while maintaining optimal epitope exposure. By integrating modern bioinformatics tools with a robust understanding of MTB pathogenesis and immune responses, this research may have the potential to significantly impact public health outcomes in the ongoing fight against TB.

## 2. Materials and Methods

### 2.1. Antigen Selection for MTB Vaccine Development

Developing a tuberculosis vaccine faces several challenges, particularly in selecting candidate antigens from nearly 4000 protein-coding genes. In this study, to overcome this bottleneck, we selected antigens Ag85A, Ag85B, Rv0288, Rv1813c, and Rv2031c for the CP91110P vaccine based on their phase-specific expression and immunogenicity, covering both latent and active phases of MTB. The amino acid sequences of these antigens were retrieved in FASTA format from the Mycobrowser database (https://mycobrowser.epfl.ch/, accessed on 25 December 2024), which provides comprehensive genomic, proteomic, drug, and transcriptomic data for pathogenic mycobacteria [28].

### 2.2. Identification and Selection of Immunodominant Helper T Lymphocyte (HTL) and Cytotoxic T Lymphocyte (CTL) Epitopes

During MTB infection, cytotoxic T lymphocytes (CTLs) play a major role in eliminating infected cells, while helper T lymphocytes (HTLs) provide auxiliary and regulatory functions. To enhance immune responses, vaccine design incorporates HTL and CTL epitopes with broad cross-reactivity [18,29,30,31]. To systematically identify immunodominant HTL and CTL epitopes derived from MTB antigens, we employed a multi-step bioinformatics pipeline integrating MHC binding prediction tools and antigenicity validation, as detailed below. First, HTL epitope candidates were predicted using the MHC-II Binding Predictions tool (http://tools.iedb.org/mhcii/, NetMHCIIpan 4.1 EL algorithm, accessed on 17 December 2024) from the Immune Epitope Database (IEDB) [32,33,34,35]. Given that MHC-II molecules typically present 12–18 amino acid (aa)-length peptides, we restricted our search to this range. Epitopes were prioritized based on strong MHC-II binding affinity, defined by a percentile rank < 0.5 (a threshold indicating that the candidate peptide binds more strongly than 99.5% of random peptides in the reference dataset). Next, CTL epitope candidates were identified using the MHC-I Binding Predictions tool (http://tools.iedb.org/mhci/, NetMHCPan 4.1 EL algorithm, accessed on 17 December 2024) from the same database. MHC-I molecules primarily present 9–10 aa peptides, so we limited our search to this length range. Similarly, high-affinity MHC-I binders were selected using a percentile rank < 0.5 cutoff to ensure robust peptide-MHC-I interaction potential. To further validate the immunogenicity of these candidates, we applied antigenicity prediction to both HTL and CTL epitope sets using the VaxiJen v2.0 database (https://www.ddg-pharmfac.net/vaxijen/VaxiJen/VaxiJen.html, accessed on 20 December 2024) [36,37]. This tool employs machine learning to estimate the potential of peptides to induce an immune response. We retained only those epitopes with an antigenicity score ≥ 0.5, a threshold validated as effective for distinguishing antigenic from non-antigenic peptides in prior studies.

Additionally, the ability of HTL epitopes to induce interferon-gamma (IFN-γ), interleukin-4 (IL-4), and IL-10 secretion was assessed using the IFNepitope (https://webs.iiitd.edu.in/raghava/ifnepitope/predict.php, accessed on 23 December 2024), IL4pred (https://webs.iiitd.edu.in/raghava/il4pred/predict.php, accessed on 23 December 2024), and IL-10Pred (https://webs.iiitd.edu.in/raghava/il10pred/predict3.php, accessed on 23 December 2024) databases [38]. To enhance T helper (Th) 1-type immune responses, HTL epitopes that induce IFN-γ secretion while suppressing IL-4 and IL-10 were prioritized. CTL epitope immunogenicity was evaluated using the IEDB Class I Immunogenicity tool (http://tools.iedb.org/immunogenicity/, accessed on 23 December 2024), which scores peptide-MHC complexes as positive (>0, indicating higher T-cell recognition likelihood and immunogenicity) or negative (<0). Epitopes with scores > 0 were selected [39]. Finally, the allergenicity and toxicity of HTL and CTL epitopes were assessed using AllerTOP v2.1 (https://www.ddg-pharmfac.net/allertop_test/, accessed on 23 December 2024) and ToxinPred (https://webs.iiitd.edu.in/raghava/toxinpred/multi_submit.php, accessed on 23 December 2024) databases [40,41,42], selecting non-allergenic and non-toxic T-cell epitopes.

### 2.3. Screening of Linear B-Cell Epitopes

B cells function by producing specific antibodies during MTB infection. Linear B-cell epitopes were identified using the ABCpred server (https://webs.iiitd.edu.in/raghava/abcpred/ABC_submission.html, accessed on 25 December 2024) with a threshold of 0.51 and an epitope length of 16 amino acid residues [43,44]. Consistent with T-cell epitope screening, allergenicity and toxicity of the epitopes were further evaluated using AllerTOP v2.1 and ToxinPred databases [40,41,42], selecting non-allergenic and non-toxic B-cell epitopes.

### 2.4. Construction of Multi-Epitope Vaccine (MEV) Candidates

Using bioinformatics tools, immunodominant HTL, CTL, and B-cell epitopes were selected to construct MEV candidates. Epitopes were linked using AAY, GPGPG, and KK linkers known for their flexibility and proteolytic resistance to enhance vaccine stability. To further boost immunogenicity, TLR-2 agonist PorB [45,46] and TLR-4 agonist heparin-binding haemagglutinin (HBHA) [47] were added to the N- and C-termini of the vaccine sequence. PorB, a TLR-2 ligand-based adjuvant, exhibits immunomodulatory properties [48], and it can activate innate immunity via the TLR2/MyD88 pathway and induce a broad adaptive immune response (Th1/Th2 antibodies, CD4^+^/CD8^+^ T cells), as well as promote antigen presentation and lymphocyte activation [49]. HBHA has strong immunostimulatory potential, and as a TLR4 agonist, it activates innate immune pathways, enhances DC maturation and antigen presentation, and promotes Th1 immune responses (IFN-γ secretion) and CTL reactions [50]. Additionally, the auxiliary peptide PADRE [46,47,51], which acts as a universal HLA-DR binder, was appended after HBHA at the C-terminus to enhance immune responses, promote CTL activity, and improve vaccine universality. A 6 × His tag was included at the end of the MEV sequence for purification and subsequent detection. Moreover, to prevent the vaccine from inducing cross-immune reactions against human proteins and avoid triggering autoimmune diseases, we used the BLASTp module of the BLAST server (https://blast.ncbi.nlm.nih.gov/Blast.cgi, accessed on 3 August 2025) to screen for homology between the vaccine and human proteins, thereby reducing the risk of autoimmunity [52,53,54,55].

### 2.5. Biological Characterization of MEV Candidates

Post-construction, the biological properties of MEV candidates critical for preclinical validation, including antigenicity, immunogenicity, allergenicity, and toxicity, were assessed using bioinformatics tools. Antigenicity was predicted using VaxiJen v2.0 and ANTIGENpro (http://scratch.proteomics.ics.uci.edu/, accessed on 25 December 2024); immunogenicity was evaluated via the IEDB server; allergenicity was detected using AllergenFP v1.1 (https://ddg-pharmfac.net/AllergenFP/, accessed on 25 December 2024) and AllerTOP v2.1; and toxicity was predicted using ToxinPred.

### 2.6. Physicochemical Properties and Solubility Assessment

The physicochemical properties of MEV candidates, critical for safety and efficacy, were assessed using the Expasy ProtParam server (https://web.expasy.org/protparam/, accessed on 25 December 2024) [56]. Parameters included amino acid count, molecular weight, isoelectric point (pI), in vitro and in vivo half-life, instability index, aliphatic index, and grand average of hydropathy (GRAVY). Vaccine solubility, essential for bioavailability and effective immune response, was evaluated using the protein-sol server (https://protein-sol.manchester.ac.uk/, accessed on 25 December 2024). This server predicts solubility by integrating 10 key features of the protein sequence, including composition, charge characteristics, and structural propensity, using a linear model [57]. A predicted solubility (QuerySol) greater than the population average (PopAvrSol = 0.45) indicated favorable solubility.

### 2.7. Global Population Coverage Analysis of HLA Alleles

HLA alleles exhibit high polymorphism, leading to variable immune responses across populations. To maximize coverage, we analyzed the global population coverage of candidate epitopes using the IEDB Population Coverage tool (http://tools.iedb.org/population/, accessed on 25 December 2024), which calculates the proportion of individuals likely to respond to a given epitope set based on HLA genotype frequencies and MHC binding data [58]. HLA genotype frequencies were sourced from the Allele Frequency Database (http://www.allelefrequencies.net/, accessed on 2 August 2025), covering 115 countries, 21 ethnic groups, and 16 geographic regions.

### 2.8. Prediction of Secondary and Tertiary Structures

The accurate prediction of secondary and tertiary structures is critical for MEV, as these structural features directly determine the proper conformational presentation of antigenic epitopes to immune cells (e.g., T cell receptors or MHC molecules). To comprehensively characterize the structural properties of the designed multi-epitope constructs, we employed a combination of well-validated bioinformatics tools for secondary structure prediction and tertiary structure modeling with refinement.

For secondary structure prediction, two complementary methods were applied: (i) PSIPRED 4.0 (http://bioinf.cs.ucl.ac.uk/psipred/, accessed on 26 December 2024), a widely used machine-learning-based tool that predicts secondary structure elements (α-helices, β-strands, and coils) by integrating sequence-derived features and evolutionary information [59]; and (ii) SOPMA (Self-Optimized Prediction Method with Alignment; https://npsa.lyon.inserm.fr/cgi-bin/npsa_automat.pl?page=/NPSA/npsa_sopma.html, accessed on 26 December 2024), which leverages homology-based alignment to predict secondary structure with high accuracy, particularly for sequences with known structural homologs [60,61,62,63,64]. These tools were selected to cross-validate predictions, ensuring robustness in defining secondary structure motifs critical for epitope accessibility and vaccine stability.

For tertiary structure prediction and optimization, we utilized AlphaFold 3 (https://alphafoldserver.com/, accessed on 26 December 2024), a state-of-the-art deep learning platform that generates high-resolution 3D protein models by predicting pairwise residue distances and orientations with unprecedented accuracy [65]. To further refine the AlphaFold 3-predicted models and improve their structural plausibility, we employed the GalaxyRefine module within the GalaxyWEB server (https://galaxy.seoklab.org/cgi-bin/submit.cgi?type=REFINE, accessed on 26 December 2024). GalaxyRefine uses molecular dynamics simulations and energy minimization to optimize local structure geometry (e.g., bond lengths, angles, and dihedrals) while preserving global fold integrity [66,67].

### 2.9. Validation of 3D Structural Models

Post-prediction and optimization, the reliability and accuracy of MEV 3D structures were validated using ProSA-web (https://prosa.services.came.sbg.ac.at/prosa.php, accessed on 30 December 2024) [68,69], ERRAT (https://saves.mbi.ucla.edu/, accessed on 30 December 2024) [70], and Structure Assessment service (https://swissmodel.expasy.org/assess, accessed on 31 December 2024) [71]. Ramachandran plots generated by the latter service assessed protein conformational reasonableness. MolProbity was used to evaluate the stereochemical rationality and atomic packing quality of the protein structure models, directly reflecting the local geometric accuracy of the structural models [72,73,74]. QMEANDisCo represented the global similarity between the computed models and the true structures, essentially serving as a model reliability score [75,76].

### 2.10. Conformational B-Cell Epitope Prediction

Conformational B-cell epitopes, which rely on antigen tertiary structure for recognition by B cell receptors, are critical for inducing high-affinity antibody responses. Notably, these epitopes constitute approximately 90% of all naturally occurring B-cell epitopes, underscoring their pivotal role in humoral immunity. To accurately identify such epitopes within the designed multi-epitope constructs, we employed the ElliPro tool (http://tools.iedb.org/ellipro/, accessed on 4 January 2025), a well-validated platform for predicting conformational B-cell epitopes based on protein structure and sequence information [77]. ElliPro integrates sequence-derived features with structural modeling to assess epitope accessibility and structural conservation, making it particularly suited for analyzing non-linear, context-dependent epitopes. Following established guidelines and prior validation studies [15,18], we applied a threshold of >0.693 for epitope inclusion. This threshold was selected based on its demonstrated ability to balance sensitivity and specificity in identifying functionally relevant conformational epitopes, ensuring that only robustly predicted candidates were retained for downstream analysis.

### 2.11. Disulfide Engineering for Enhanced Structural Stability

To improve the stability and proper folding of the MEV—an essential requirement for maintaining antigenic integrity and immunogenicity—we implemented disulfide engineering using Disulfide by Design 2.0 (DbD2; http://cptweb.cpt.wayne.edu/DbD2/, accessed on 4 January 2025) [78]. Disulfide bonds, formed through oxidative pairing of cysteine residues, are powerful modulators of protein structure, enhancing thermostability, resistance to proteolysis, and correct folding kinetics [78,79]. DbD2 is a computational tool specifically designed to predict optimal cysteine residue pairs for introducing disulfide bonds in target proteins. It evaluates potential cysteine-cysteine interactions by calculating key structural parameters, including the Chi3 torsion angle (a measure of side-chain conformational flexibility), bond energy (reflecting the strength of the disulfide linkage), and ΣB-factor values (indicative of atomic displacement and structural disorder). By prioritizing pairs with favorable Chi3 angles, low (more negative) bond energies (denoting stronger covalent interactions), and reduced ΣB-factors (indicating greater structural rigidity), we aimed to engineer disulfide bonds that would stabilize the tertiary structure of the MEV without disrupting critical epitope conformations.

### 2.12. Molecular Docking with TLR-2 and TLR-4

To enhance immune responses by activating innate immune signaling pathways through TLR targeting, we performed molecular docking simulations between the MEV construct and key TLRs: TLR-2 (PDB ID: 5D3I) and TLR-4 (PDB ID: 2Z65). These TLRs were selected based on their well-documented roles in recognizing bacterial components and their relevance to anti-MTB immunity [5,9,80]. Structural coordinates for TLR-2 and TLR-4 were retrieved from the Protein Data Bank (PDB; https://www.rcsb.org/).

Molecular docking was conducted using a two-step workflow: First, the MEV structure—predicted previously using AlphaFold 3 (https://alphafoldserver.com/) for high-confidence modeling—was prepared as a ligand, with its structure optimized to ensure geometric and energetic plausibility. Second, rigid-body docking simulations were performed using ClusPro 2.0 (https://cluspro.bu.edu/home.php, accessed on 6 January 2025), a widely validated automated docking server that employs a coarse-grained potential to efficiently explore binding poses and select energetically favorable interactions [81,82,83,84,85]. This tool was chosen for its robustness in predicting protein-protein docking interfaces, particularly for receptor-ligand pairs with moderate to high affinity.

Post-docking analysis focused on identifying key interaction hotspots, including hydrogen bonds, hydrophobic contacts, and electrostatic interactions. The top-ranked docking model (selected based on ClusPro’s built-in scoring function) was visualized in 3D using PyMOL (version 3.1.3, https://pymol.org/2/, accessed on 6 January 2025), enabling detailed inspection of epitope-receptor contacts and conformational alignment. Additionally, 2D interaction diagrams were generated using LigPlot (https://www.ebi.ac.uk/thornton-srv/software/LigPlus/, accessed on 7 January 2025), which graphically summarizes residue-level interactions (e.g., bond distances, angles, and types) to highlight critical binding determinants.

### 2.13. Normal Mode Analysis (NMA) of TLR Complexes

To characterize the biophysical properties of the MEV-TLR complexes and evaluate their stability and conformational dynamics—key determinants of signaling activation—we applied Normal Mode Analysis (NMA) using the iMODS web server (https://imods.iqf.csic.es/, accessed on 9 January 2025) [86,87,88]. NMA, a computational method that models molecular motion through low-frequency vibrational modes in internal (torsion) coordinates, is particularly valuable for assessing structural flexibility and rigidity in protein complexes.

For this analysis, the optimized 3D structures of MEV-TLR-2 and MEV-TLR-4 complexes (derived from the docking simulations) were input as PDB-formatted files. iMODS computed a range of dynamic parameters, including: (i) flexibility profiles, which identify regions of high or low mobility (critical for understanding ligand accessibility and conformational changes during signaling); (ii) B-factor values, experimental measures of atomic displacement derived from X-ray crystallography, here used to validate predicted flexibility; (iii) eigenvalues and variance, which quantify the amplitude of motion for each normal mode; (iv) covariance matrices, illustrating correlated motions between residues; and (v) elastic network models, which approximate the complex as a network of springs to predict how structural perturbations propagate through the system. By integrating these metrics, NMA provided insights into the stability of the MEV-TLR interfaces and the potential for induced conformational changes upon binding—features critical for activating downstream innate immune signaling cascades (e.g., NF-κB or IRF3 pathways). This analysis ensured that the selected MEV-TLR complexes were not only structurally compatible but also dynamically capable of inducing the desired immunostimulatory responses.

### 2.14. Molecular Dynamics (MD) Simulation Analysis

MD simulations were performed using the Desmond module (Schrödinger Suite) to evaluate the stability and dynamic interactions of protein-ligand complexes. The system was prepared via Maestro’s Protein Preparation Wizard, solvated in an orthorhombic SPC water box, and neutralized with Na^+^/Cl^−^ ions under physiological conditions (0.15 M NaCl). Energy minimization and 200 ns simulations under the NPT ensemble (300 K, 1 atm) employed the OPLS4 force field. Short-range electrostatic interactions were calculated using the particle mesh Ewald method (9.0 Å cutoff). Temperature and pressure were regulated via the Nosé-Hoover thermostat and Martyna-Tuckerman-Klein barostat, respectively. Trajectories (saved at 100 ps intervals) were analyzed for root mean square deviation (RMSD), root mean square fluctuation (RMSF), and radius of gyration (Rg) using GROMACS after format conversion with MDTraj.

Principal component analysis (PCA) was conducted by diagonalizing the covariance matrix of C-alpha atomic coordinates derived from MD trajectories. Dominant motion modes were identified via eigenvalues and eigenvectors, with projections generated using GROMACS 2025.1 tools (gmx covar, gmx anaeig). Free energy landscapes (FELs) were calculated using Gibbs free energy (gmx sham) and visualized against PC1 and PC2. LOESS regression and KDE validated FEL clarity. Dynamic cross-correlation matrices (DCCMs) were computed with Bio3D (R environment) after trajectory conversion to DCD format, quantifying residue motion correlations (*C*(*i*,*j*)) based on C-alpha displacement vectors (Δ*ri*).

$$C(i,j)=\frac{\left(\Delta ri\times \Delta rj\right)}{{\left({\left|\Delta ri\right|}^{2}\right)}^{\frac{1}{2}}{\times ({\left|\Delta rj\right|}^{2})}^{\frac{1}{2}}}$$

### 2.15. Immune Response Simulation Using C-ImmSim

To predict the in vivo immunogenicity of the MEV construct prior to experimental validation, we employed the C-ImmSim server (https://150.146.2.1/C-IMMSIM/, accessed on 13 January 2025) [89,90,91], an agent-based modeling platform that simulates dynamic interactions between immune cells and pathogen-derived antigens. This simulation aimed to evaluate the potential efficacy of three vaccine administration timepoints (days 1, 84, and 168), mimicking a typical prime-boost-boost immunization regimen. C-ImmSim integrates key components of innate and adaptive immunity, including natural killer (NK) cells, macrophages, dendritic cells (DCs), B cells, HTLs, CTLs, antibody-secreting plasma cells, and a spectrum of cytokines (e.g., IL-2, IFN-γ, tumor necrosis factor (TNF)-α). Default parameters were applied, including a simulation volume of 50 (representing a standardized tissue microenvironment), 1100 simulation steps (capturing immune dynamics over a biologically relevant timeframe), and a random seed of 1234 (ensuring reproducibility).

### 2.16. Codon Optimization and Recombinant Plasmid Design

To enhance heterologous expression of the MEV construct in *Escherichia coli* (*E. coli*) strain 536, we performed codon optimization using the Optimizer tool (http://genomes.urv.es/OPTIMIZER/, accessed on 15 January 2025) [92]. Codon usage bias—defined as the non-random preference for specific codons encoding the same amino acid—significantly impacts translation efficiency and protein yield in heterologous hosts. Thus, the optimizer was configured to maximize the codon adaptation index (CAI ≈ 1), a metric reflecting how well the target sequence matches the host’s codon usage preferences, while maintaining GC content within the optimal range (30–70%) to minimize mRNA secondary structure formation and AT content to avoid regions prone to translation pausing.

The optimized MEV coding sequence was cloned into the prokaryotic expression vector pET28a(+), which contains a T7 promoter for high-level transcription and a C-terminal His-tag for affinity purification. Restriction enzyme sites XhoI and BamHI were selected for cloning based on their compatibility with the MEV sequence and the multiple cloning site (MCS) of pET28a(+). To verify the cloning strategy, we used SnapGene 8.0.0 software to simulate digestion of the recombinant plasmid with XhoI/BamHI, generating a 1% agarose gel electrophoresis profile.

## 3. Results

### 3.1. Identification of Immunodominant Epitopes, MEV Design, and Population Coverage

**Figure 1:**
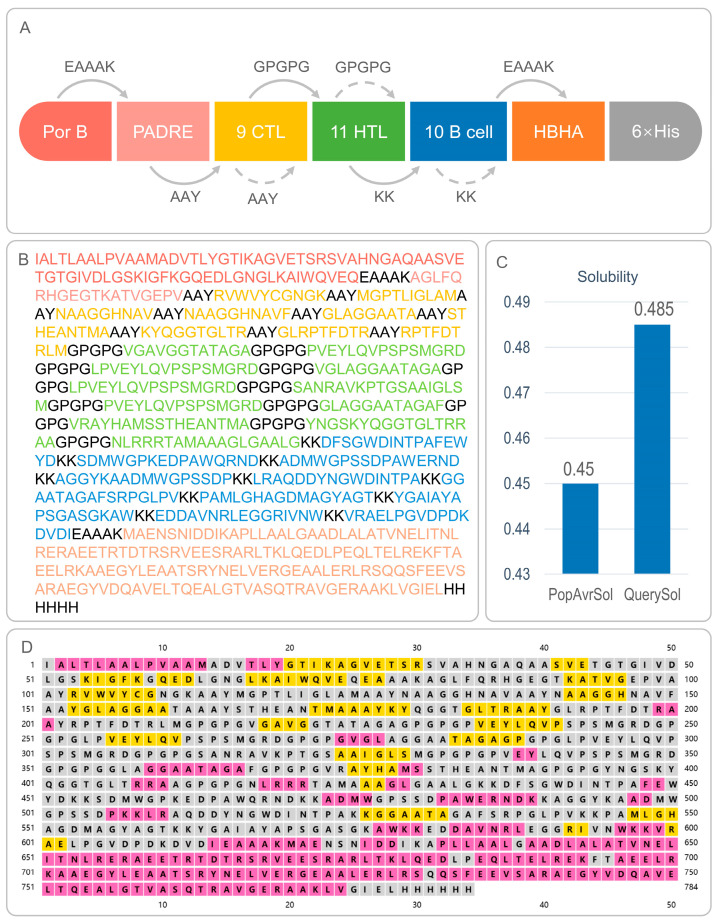
Composition, amino acid sequence, solubility, and secondary structure of CP91110P. (**A**) Schematic representation of CP91110P components: TLR2 agonist PorB, helper peptide PADRE, nine cytotoxic T lymphocyte (CTL) epitopes, 11 helper T lymphocyte (HTL) epitopes, ten B-cell epitopes, and TLR4 agonist HBHA. (**B**) Amino acid sequence of CP91110P with color-coded components. (**C**) Solubility analysis using Protein-Sol server (threshold: >0.45). (**D**) Secondary structure prediction via PSIPRED: α-helices (pink), β-strands (yellow), and random coils (gray).

Eleven immunodominant HTL epitopes, nine CTL epitopes, and ten B-cell epitopes were identified from the epitopes of five MTB antigens (Figure 1A, Table 1 and Table S1). All epitopes exhibit favorable percentile ranks, antigenicity, immunogenicity, and non-toxicity/non-allergenicity, which predict strong T/B cell activation. These T-cell and B-cell epitopes were cross-validated in the IEDB database and found to be unverified epitopes. Epitopes were linked via AAY, GPGPG, and KK spacers, with TLR-2 agonist PorB, TLR-4 agonist HBHA, and PADRE adjuvant integrated using EAAAK linkers (Figure 1B). The homology between the vaccine epitopes and the human proteome was checked using BLASTp, and the results showed that the vaccine epitopes were non-homologous to human proteins. The MEV construct, CP91110P, exhibited 86.18% global population coverage for HLA-I/II alleles, suggesting broad potential applicability across major ethnic groups (Table 2).

### 3.2. Antigenicity, Immunogenicity, and Safety Profile of CP91110P

CP91110P exhibited favorable immunological and biophysical properties that suggest its potential as a vaccine candidate (Table 3, Figure 1C): (1) Antigenicity and immunogenicity. Quantitative assessment revealed elevated antigenic potential, with VaxiJen v2.0 and ANTIGENpro scores of 0.8789 and 0.801088, respectively—both exceeding typical thresholds (generally >0.5–0.6) for identifying promising vaccine antigens. Complementary immunogenicity analysis using the IEDB database further indicated its capacity to induce adaptive immune responses, yielding an immunogenicity score of 4.40091, consistent with strong peptide-MHC binding and T cell activation potential. (2) Biophysical stability. CP91110P (784 residues; 80.7 kDa) exhibited favorable biophysical characteristics for stability and expression. Its isoelectric point (pI 7.38) was near-neutral, potentially minimizing non-specific charge-based aggregation. The instability index (33.48)—calculated to assess proteolytic susceptibility—fell well below the threshold (≤40) for soluble expression, indicating thermodynamic stability. Additionally, a solubility score of 0.485 (exceeding the 0.45 empirical threshold) suggested efficient cytoplasmic expression and reduced inclusion body formation in heterologous hosts. (3) In silico pharmacokinetic projection. In silico half-life predictions highlighted prolonged in vivo persistence: CP91110P displayed a half-life of 20 h in mammalian reticulocytes (relevant for therapeutic protein applications) and >10 h in *E. coli* (a key host for recombinant expression). These extended half-lives are advantageous for maintaining stable protein levels in vivo.

### 3.3. Secondary and Tertiary Structural Analysis of CP91110P

**Figure 2:**
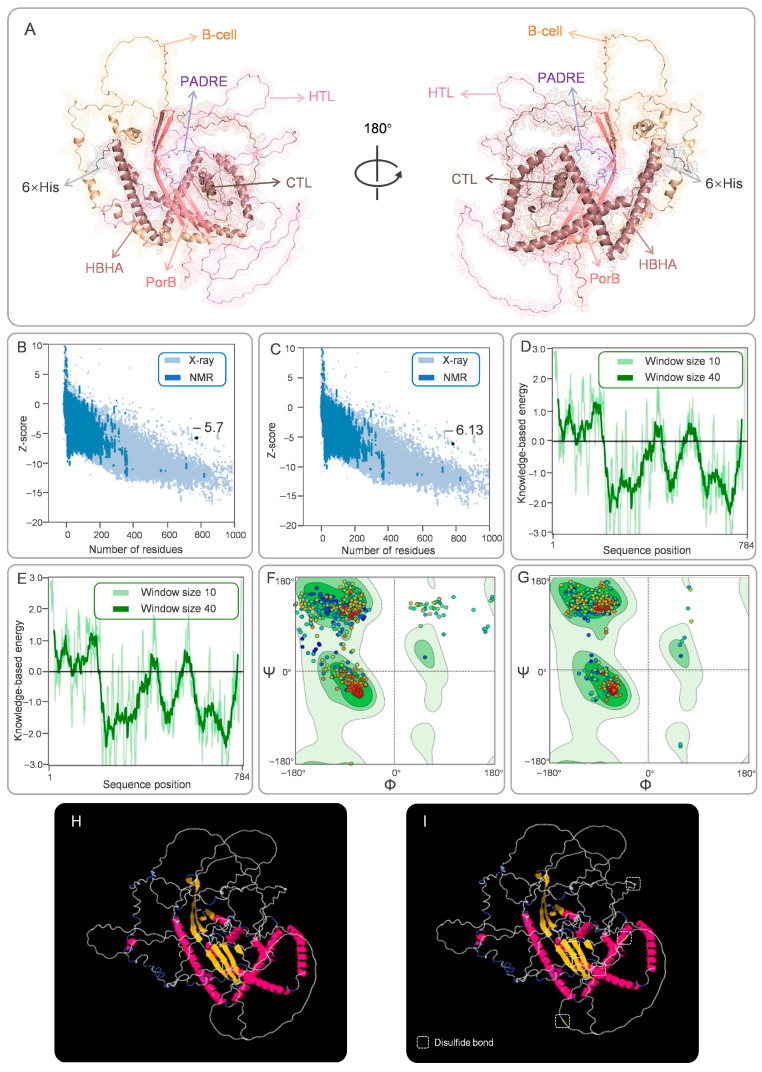
Tertiary structure construction, refinement, conformational B-cell epitopes, and disulfide engineering of CP91110P. (**A**) Optimized tertiary structure model generated by Galaxy Refinement and visualized in PyMOL (color-coded components). (**B**,**C**) ProSA-web Z-scores (−5.7 pre-optimization; −6.13 post-optimization). (**D**,**E**) ERRAT validation of tertiary structure energy profiles. (**F**,**G**) Ramachandran plots: favored (dark green), allowed (light green), and disallowed (white) regions. In a Ramachandran plot, each point represents an amino acid residue. The color gradient from dark blue to red indicates the sequence direction from the N-terminus to the C-terminus. (**H**,**I**) Disulfide-engineered structure (yellow bars indicate introduced bonds) compared to the native conformation.

The structural characteristics of CP91110P were comprehensively analyzed using a combination of bioinformatics tools to evaluate both secondary and tertiary structural features, critical for understanding its functional potential. (1) Secondary structure composition. Secondary structure prediction using PSIPRED (a neural network-based algorithm) and SOPMA (a homology-based statistical method) revealed a conserved structural motif in CP91110P. The analysis predicted a secondary structure composition of 23.47% α-helices, 16.07% β-strands, and 60.46% random coils (Figure 1D). This distribution is consistent with typical globular proteins, where random coils dominate flexible regions, while α-helices and β-strands contribute to structural stability. (2) Tertiary structure modeling and optimization. For tertiary structure resolution, an initial model was generated using AlphaFold3, a state-of-the-art deep learning-based tool for high-accuracy protein structure prediction. This initial model was further refined using the GalaxyWEB server’s optimization module, yielding an optimized model (designated Model 5; Figure 2A). Key structural reliability metrics were significantly improved following optimization:

Z-score: Increased from −5.7 to −6.13 (higher Z-scores indicate better model quality relative to a dataset of known structures; Figure 2B,C).

ERRAT quality factor: Improved from 87.04 to 89.97 (scores > 85 are considered indicative of high structural reliability; Figure 2D,E).

Ramachandran plot analysis: The proportion of residues in favorable backbone conformations increased from 82.48% (initial model) to 98.34% (optimized Model 5; Figure 2F,G). This marked improvement suggests that the refined model better captures the native-like geometry of CP91110P, with minimal steric clashes or unfavorable torsion angles. The MolProbity Score decreased from 2.13 to 0.66, with a value below 1.00 indicating a significant improvement in the quality of the vaccine structure model. The QMEANDisCo score slightly increased from 0.25 to 0.29. Since CP91110P is not a natural protein, the lower score is expected.

### 3.4. Conformational B-Cell Epitopes and Disulfide Bond Engineering

**Figure 3:**
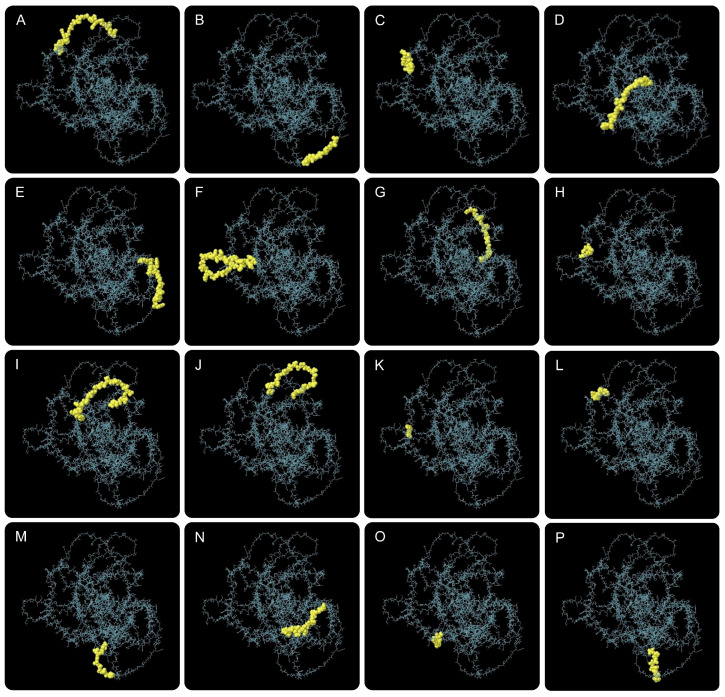
Conformational B-cell epitopes predicted in CP91110P by ElliPro. Sixteen potential epitopes (panel (**A**–**P**), yellow spheres) mapped onto the tertiary structure (gray backbone).

To characterize the humoral immune recognition potential of CP91110P and enhance its structural stability, we analyzed conformational B-cell epitopes and engineered stabilizing disulfide bonds. (1) Conformational B-Cell epitope identification. Using ElliPro, a structure-based B-cell epitope prediction tool, we identified 16 conformational B-cell epitopes across the CP91110P structure. These epitopes, defined by their spatial accessibility and structural conservation, displayed high confidence scores ranging from 0.694 to 0.926 (with scores >0.5 indicating robust prediction reliability; Figure 3, Table S2). This result suggests the presence of multiple distinct antigenic regions that may drive high-affinity antibody responses. (2) Disulfide bond engineering for stability enhancement. To improve structural stability, we applied DbD2 and six optimal disulfide bonds were introduced, each characterized by favorable dihedral angles (χ3) and low bond energies (Figure 2H,I, Table S3), which are indicative of strong covalent linkages:

GLY56-GLU74: χ3 = −67.43°, energy = 1.38 kcal/mol
THR183-GLY184: χ3 = +93.67°, energy = 1.14 kcal/mol
GLU384-ALA385: χ3 = +95.83°, energy = 0.82 kcal/mol
PRO393-GLY394: χ3 = +95.99°, energy = 1.35 kcal/mol
ALA425-ALA426: χ3 = +96.76°, energy = 1.77 kcal/mol
GLY638-LEU717: χ3 = +78.50°, energy = 1.88 kcal/mol

### 3.5. High-Affinity Binding of CP91110P to TLR-2 and TLR-4

**Figure 4:**
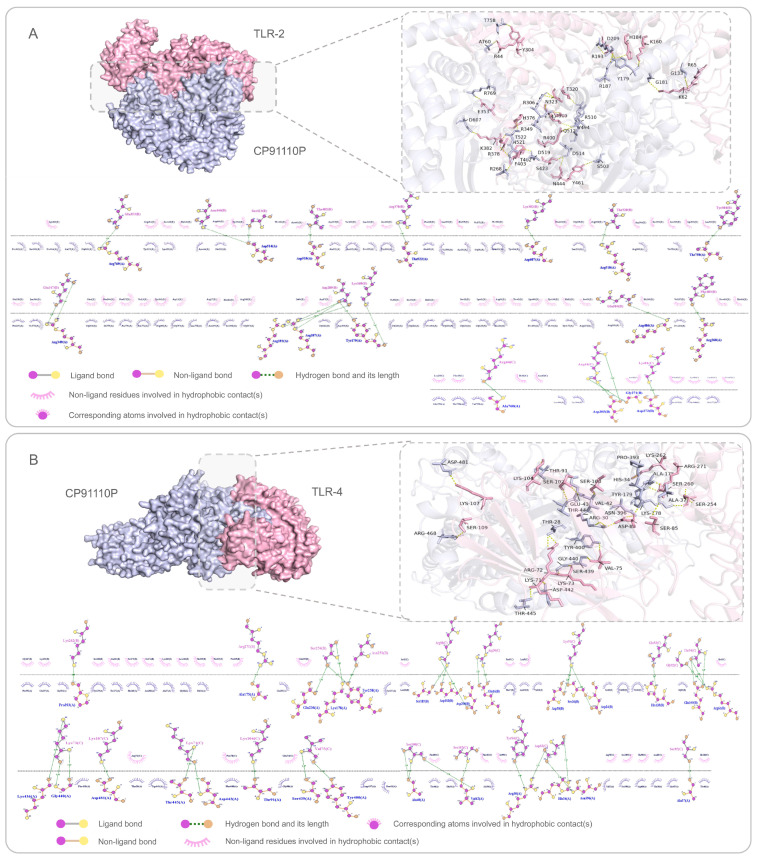
Molecular docking and interaction analysis of CP91110P with TLR-2 (**A**) and TLR-4 (**B**). Predicted 3D interactions (AlphaFold-3) and 2D ligand-receptor diagrams (LigPlot). CP91110P (purple) binds TLR-2 (pink, **A**) and TLR-4 (pink, **B**). Insets: Enlarged views of key residue interactions (hydrogen bonds, hydrophobic contacts, ligand/non-ligand bonds).

To evaluate the interaction strength between CP91110P and key innate immune receptors (TLR-2 and TLR-4), we performed molecular docking simulations using AlphaFold 3 for target structure prediction and ClusPro 2.0 for complex modeling. These analyses suggested robust binding affinities, with calculated binding free energies (ΔG) of −1535.9 kcal/mol for the CP91110P-TLR-2 complex and −1672.5 kcal/mol for the CP91110P-TLR-4 complex. The more negative ΔG value for TLR-4 may indicate stronger thermodynamic stabilization of this interaction. Further structural analysis using LigPlot highlighted the contribution of intermolecular hydrogen bonds to these interactions. The CP91110P-TLR-2 complex formed 22 hydrogen bonds, while the CP91110P-TLR-4 complex exhibited 37 hydrogen bonds (Figure 4A,B). This higher number of hydrogen bonds in the TLR-4 complex may likely underpin its stronger binding affinity, as hydrogen bonding is a critical driver of molecular recognition and stability in protein–protein interactions.

### 3.6. Dynamic Behavior and Flexibility of CP91110P-TLR Complexes

**Figure 5:**
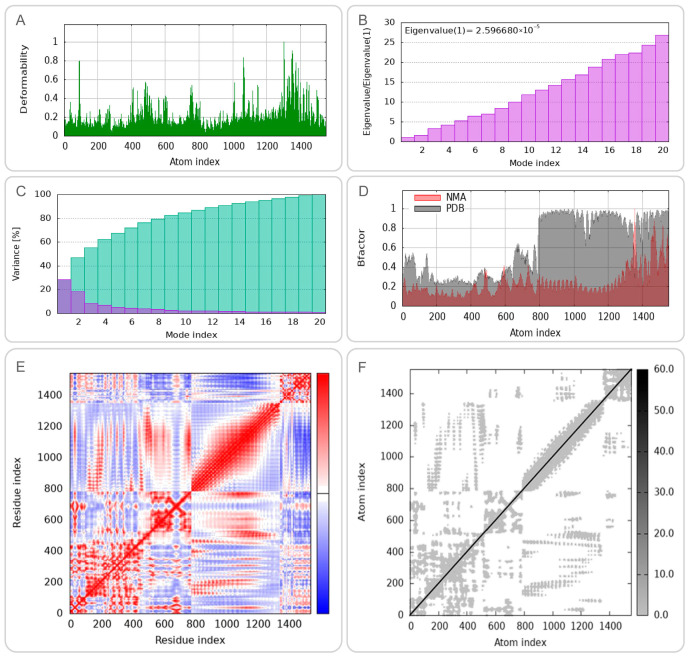
Normal mode analysis (NMA) of CP91110P-TLR-2 complex (iMODS). (**A**) Deformability, (**B**) eigenvalues, (**C**) variance per mode index, (the variance associated to each normal mode is inversely related to the eigenvalue. Colored bars show the individual [purple] and cummulative [green] variances), (**D**) B-factor distribution, (**E**) covariance matrix (red: correlated; blue: anti-correlated motions), (**F**) elastic network model (dark gray: rigid regions).

**Figure 6:**
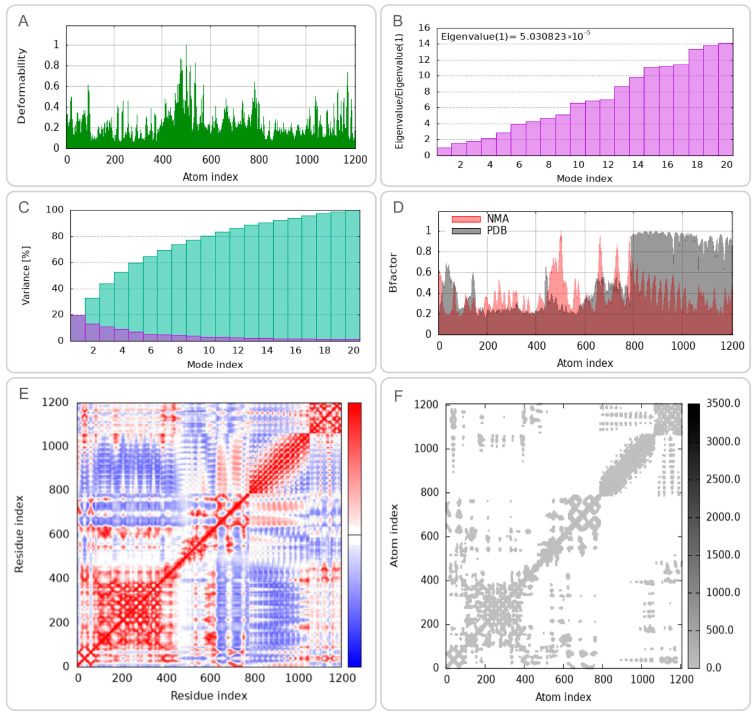
Normal mode analysis (NMA) of CP91110P-TLR-4 complex (iMODS). (**A**) Deformability, (**B**) eigenvalues, (**C**) variance per mode index, (the variance associated to each normal mode is inversely related to the eigenvalue. Colored bars show the individual [purple] and cummulative [green] variances), (**D**) B-factor distribution, (**E**) covariance matrix, (**F**) elastic network model.

To characterize the conformational dynamics and mechanical properties of the CP91110P-TLR-2 and CP91110P-TLR-4 complexes, we performed NMA using the iMODS web server. (1) Deformation peaks and structural rigidity: Both complexes exhibited distinct deformation peaks, representing regions of high mobility (Figure 5A and Figure 6A). Quantitative analysis of eigenvalues—metrics inversely related to structural stiffness—revealed higher rigidity in the CP91110P-TLR-4 complex (eigenvalue = 5.030823 ×10^−5^) compared to the TLR-2 complex (eigenvalue = 2.596680 × 10^−5^). A lower eigenvalue indicates greater resistance to deformation, suggesting that the TLR-4 complex maintains a more rigid conformation under physiological conditions. (2) Cumulative variance and major motion modes: Cumulative variance analysis, which quantifies the proportion of total motion explained by the first n modes, identified mode indices 9 (TLR-2) and 10 (TLR-4) as capturing >80% of the total conformational dynamics (Figure 5C and Figure 6C). This indicates that the primary functional motions of both complexes are driven by a small subset of low-frequency modes, consistent with the idea that TLRs and their ligands undergo coordinated, collective movements to initiate signaling. (3) Flexibility and stability correlation: B-factor analysis, which reflects atomic displacement from X-ray crystallography data, further indicated enhanced structural stability in the NMA-refined models. Both complexes displayed reduced B-factors compared to unrefined structures (Figure 5D and Figure 6D), indicating that the optimized models better captured the native-like, low-flexibility state of the TLR-ligand interaction. (4) Correlated motions and dynamic heterogeneity: Covariance maps and elastic network models highlighted cooperative motions between residues, with TLR-4 complexes exhibiting more pronounced dynamic heterogeneity (Figure 5E,F and Figure 6E,F). This suggests that while both complexes share conserved rigid-body motions, the TLR-4 interface may retain greater flexibility in specific regions, potentially to accommodate downstream signaling events or interactions with co-receptors.

### 3.7. Structural Stability and Conformational Dynamics of CP91110P-TLR Complexes

**Figure 7:**
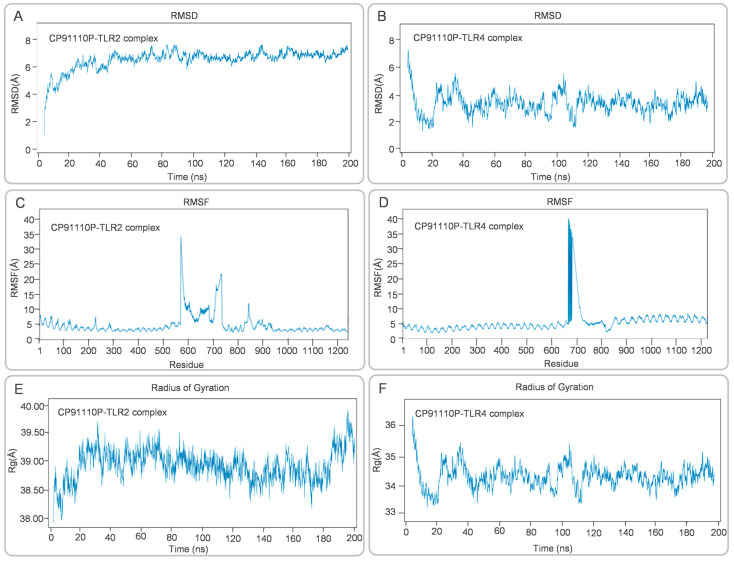
Structural dynamics of CP91110P-TLR complexes: RMSD, RMSF, and radius of gyration (Rg). (**A**,**B**) Root mean square deviation (RMSD) trajectories demonstrate stability for the TLR2 complex (6–8 Å) and moderate stability for the TLR4 complex (2–6 Å). (**C**,**D**) Root mean square fluctuation (RMSF) profiles highlighting residue-specific mobility. (**E**,**F**) Radius of gyration (Rg) values reflecting extended (TLR2, 38.50–39.50 Å) versus compact (TLR4, 33–36 Å) conformations.

MD simulations were conducted to assess the structural dynamics and stability of CP91110P-TLR complexes. RMSD analysis revealed distinct conformational behaviors between the two complexes. For the CP91110P-TLR2 complex, RMSD values gradually increased during the initial 20 ns, stabilizing at 6–8 Å for the remainder of the simulation, indicating structural reorganization followed by steady-state conformation (Figure 7A). In contrast, the CP91110P-TLR4 complex exhibited significant fluctuations during the first 40 ns, after which the RMSD values gradually converged to within the range of 2–6 Å (Figure 7B). RMSF analysis highlighted residue-specific flexibility. The RMSF analysis was performed to evaluate the flexibility of amino acid residues within the vaccine-receptor complexes over the course of the 200 ns MD simulation. The CP91110P–TLR2 complex exhibited localized mobility peaks around residues 550 and 700, which may correspond to flexible loop regions or ligand-binding interfaces, while the core structural regions remained relatively stable with minimal fluctuations (Figure 7C). Similarly, the CP91110P–TLR4 complex demonstrated a prominent peak near residue 600, suggesting the presence of a dynamic or functionally relevant domain. Nevertheless, the majority of residues in this complex also showed fluctuations below 3 Å, indicating that the system maintained overall structural stability throughout the simulation period (Figure 7D). These results suggest the conformational integrity and stable interactions of the vaccine-receptor complexes. Rg measurements further differentiated the complexes: CP91110P-TLR2 complex maintained an extended conformation (Rg 38.50–39.50 Å, Figure 7E), whereas CP91110P-TLR4 complex adopted a compact architecture (Rg 33–36 Å, Figure 7F) with initial structural adjustments.

**Figure 8:**
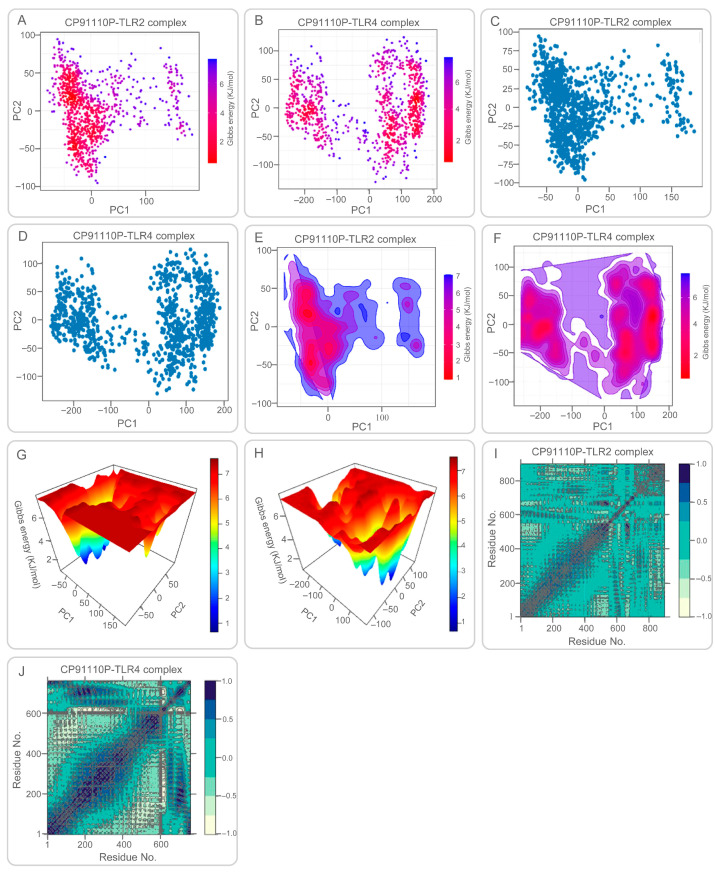
Principal component analysis (PCA), Gibbs free energy landscapes, and dynamic cross-correlation matrices (DCCM) of CP91110P-TLR complexes. (**A**–**D**) PCA and free energy landscapes: TLR2 complexes exhibit broad conformational sampling (low-energy basins), while TLR4 complexes adopt restricted states. (**E**–**H**) 2D/3D free energy surfaces illustrating structural flexibility (TLR2) versus rigidity (TLR4). (**I**,**J**) DCCM maps: TLR2 shows synchronized motions, whereas TLR4 displays localized flexibility.

PCA and FEL studies provided insights into conformational sampling. CP91110P-TLR2 complex exhibited a broad, continuous energy distribution across PC1 and PC2, reflecting structural flexibility and diverse low-energy states (Figure 8A,C,E,G). Conversely, CP91110P-TLR4 complex displayed two distinct clusters separated by an energy barrier, suggesting restricted conformational transitions (Figure 8B,D,F,H). DCCM analysis revealed synchronized motions in the CP91110P-TLR2 complex (Figure 8I), critical for structural coherence, while the CP91110P-TLR4 complex showed localized correlations, emphasizing adaptive flexibility (Figure 8J). These findings suggest the interplay between structural rigidity and adaptability in vaccine-receptor interactions, with implications for optimizing epitope presentation and immune activation.

To extend our molecular dynamics findings and evaluate the thermodynamic stability of the CP91110P–TLR complexes, we performed Molecular Mechanics Poisson–Boltzmann Surface Area (MM-PBSA) and Molecular Mechanics Generalized Born Surface Area (MM-GBSA) binding free energy calculations. These methods provide detailed estimations of binding free energies by decomposing molecular interactions into van der Waals, electrostatic, polar solvation, and non-polar solvation energy terms. Representative frames from the last 20 ns of the 100 ns MD simulation trajectories were used for the calculations.

The MM-PBSA results revealed that the CP91110P–TLR2 complex had a more favorable binding free energy (ΔGbind = −59.5 kcal/mol) than the CP91110P–TLR4 complex (ΔGbind = −46.7 kcal/mol) (Table 4). Similarly, MM-GBSA calculations showed binding free energies of −67.8 kcal/mol for TLR2 and −53.8 kcal/mol for TLR4. In both models, electrostatic (ΔEele) and van der Waals (ΔEvdw) interactions were the dominant stabilizing forces, whereas polar solvation (ΔGPB ΔGGB) had a destabilizing effect, partially compensated by favorable non-polar solvation contributions (ΔGSA) (Table 5).

These quantitative results are consistent with the dynamic behavior observed during MD simulations: the TLR2 complex maintained greater structural integrity and lower root mean square deviation (RMSD: 6–8 Å), the TLR4 complex fluctuates greatly during the first 40ns of the simulation, after which the RMSD values remain in the range of 2–6 Å. The radius of gyration (Rg) and PCA further confirmed that CP91110P adopts a more extended, stable conformation with TLR2 and a more compact, fluctuating state with TLR4. These findings support a model wherein TLR2 provides structural anchoring and high-affinity binding, while TLR4 contributes adaptive flexibility, collectively potentially enhancing immune receptor engagement and downstream immunogenic signaling.

### 3.8. CP91110P Elicits Robust Innate and Adaptive Immune Responses

**Figure 9:**
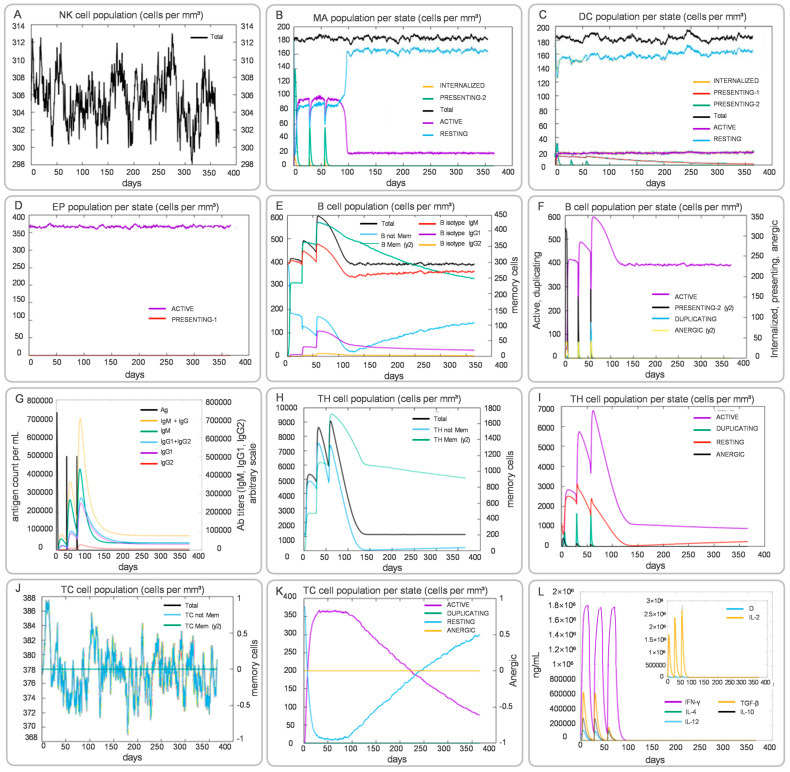
Simulated innate and adaptive immune responses induced by CP91110P (C-ImmSim). (**A**–**L**) Post-vaccination dynamics: NK cells (**A**), macrophages (**B**), DCs (**C**), epithelial cells (**D**), B-cell populations (**E**,**F**), antibody levels (**G**), helper T-cell subsets (**H**,**I**), cytotoxic T-cell subsets (**J**,**K**), and cytokine profiles (**L**).

The C-ImmSim modeling revealed that CP91110P is predicted to elicit multifaceted immune activation, encompassing both innate and adaptive responses with distinct spatiotemporal dynamics (Figure 9). In the innate immune compartment, NK cell total counts exhibited fluctuating elevations throughout the observation period, indicating persistent involvement of these sentinel lymphocytes in immune surveillance (Figure 9A). Macrophages demonstrated a striking activation profile, with the PRESENTING-2 subset peaking at days 3–5 post-vaccination, significantly surpassing levels of the RESTING state, which suggested their pivotal role in early antigen presentation and phagocytic activity (Figure 9B). DCs primarily maintained a RESTING phenotype (dominating the DC population), though this stable state may have masked underlying priming mechanisms critical for downstream adaptive responses (Figure 9C). Epithelial cells (EP population) showed minimal fluctuations, suggesting preserved tissue integrity and absence of significant immunopathology during the immune response (Figure 9D).

Adaptive immune responses unfolded with coordinated humoral and cellular components. B cell subsets displayed dynamic reconfiguration: memory B cells (B Mem, y2) expanded markedly from days 7–10, coinciding with a decline in naïve B cells (B naive), a pattern indicative of robust germinal center activity and B cell maturation (Figure 9E,F). Serum antibody profiling revealed increasing levels of IgM, with sustained high titers of IgG and its subtypes IgG1 and IgG2 throughout the immune response (Figure 9G). T cell dynamics were similarly complex: helper T cells (TH cells) underwent rapid proliferation, with the ACTIVE subset peaking on day 10 before transitioning to the memory TH Mem (y2) subset, which persisted at stable levels (Figure 9H,I). Cytotoxic T cells (TC cells) exhibited similar kinetic patterns: the effector subset (ACTIVE) showed significant early proliferation during infection, while the memory subset (TC Mem, y2) remained stable throughout the immune response, indicating a potential long-term reservoir of specific killing capacity (Figure 9J,K). Notably, TC cells lacking a memory phenotype (TC not Mem) gradually declined with fluctuations over time, reflecting the immune system’s precise regulation (Figure 9J). Cytokine profiling (Figure 9L) further elucidated a Th1-biased immune milieu, characterized by sustained high levels of IFN-γ and IL-2—key drivers of cellular immunity—peaking during days 7–14. Transient elevations in Th2-associated cytokines (e.g., IL-4, IL-6) were restricted to the early phase, followed by sharp declines, which may have prevented excessive inflammation.

### 3.9. Codon Optimization, Recombinant Plasmid Construction, and Gel Electrophoresis Validation

**Figure 10:**
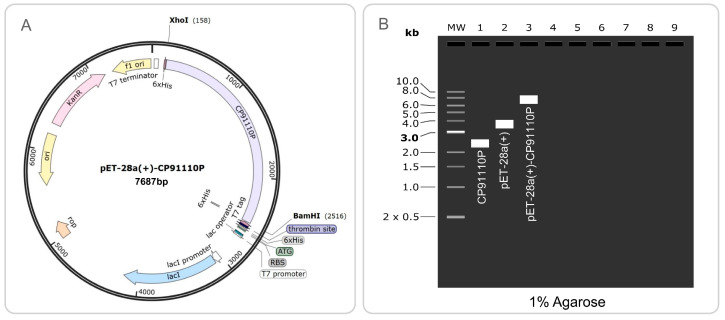
Codon optimization and recombinant plasmid construction of CP91110P. (**A**) Optimized codon sequence (CAI: 0.746; GC content: 61.4%) cloned into pET28a(+) via XhoI/BamHI restriction sites. (**B**) Simulated 1% agarose gel electrophoresis: Lane 1 (CP91110P insert), Lane 2 (pET28a(+)), Lane 3 (recombinant pET28a(+)-CP91110P).

The codon-optimized CP91110P sequence, characterized by a high codon adaptation index (CAI = 0.746) and balanced GC content (61.4%), was successfully cloned into the pET28a(+) prokaryotic expression vector using XhoI and BamHI restriction sites. The resulting recombinant plasmid (7687 bp) contained the optimized CP91110P insert spanning nucleotides 158 to 2516 of the vector backbone (Figure 10A). To verify the integrity of the cloning process, simulated 1% agarose gel electrophoresis was performed. This analysis suggested the correct assembly of the recombinant construct, with the CP91110P insert migrating at the expected size (Figure 10B), which is consistent with the predicted length of the optimized gene fragment.

## 4. Discussion

The present study designed a novel MEV, CP91110P, using computational biology approaches, aiming to target both active and latent TB infections [6,15,17,93]. The vaccine was constructed by prioritizing immunodominant epitopes from MTB antigens spanning both proliferative and latent phases, combined with TLR agonists (PorB, HBHA) and a helper peptide (PADRE) to enhance immunogenicity [6,15,17]. In silico analyses predicted favorable properties of cp91110p: high predicted antigenicity (vaxijen score: 0.8789, threshold > 0.5) and immunogenicity (iedb score: 4.40091); broad global hla-i/ii coverage (86.18%); stable tertiary structure (98.34% residues in ramachandran-favored regions); strong binding affinity to tlr-2 (−1535.9 kcal/mol) and tlr-4 (−1672.5 kcal/mol); and robust predicted immune responses, including th1 polarization (dominated by ifn-γ/il-2) and activation of innate immune cells (e.g., nk cells, macrophages and dcs). Additionally, cp91110p exhibited favorable physicochemical properties, such as low instability index (33.48), good solubility (0.485), and efficient expression potential in *E. coli* (cai: 0.746), supporting its potential for clinical translation.

BCG, currently the most widely used TB vaccine, holds specific value in TB prevention and control: it plays a crucial role in preventing disseminated TB and tuberculous meningitis in infants and young children [94,95], but its protective efficacy against adult pulmonary TB is limited [5,96,97,98]. More notably, the immune protection induced by BCG gradually wanes over time, and this issue may be addressed through the “BCG prime-boost” immunization strategy [99,100,101]. In the selection of booster vaccines, epitope vaccines exhibit unique advantages: compared with other TB vaccines, the latter often have problems such as a single antigen and insufficient epitope coverage, while epitope vaccines can bind to and be recognized by multiple MHC molecules in populations with different genetic backgrounds, ensuring the efficiency of immune presentation [9,102,103]. The design process involves steps such as selection of potential antigens, prediction and identification of immunodominant epitopes, comparison of MHC affinity, addition of adjuvants or helper peptides, codon optimization, and prediction of structure and function [6,15,16,19,20,21,104], which enables vaccines incorporating multiple immunodominant epitopes to induce stronger immune responses, while improving immune efficiency and reducing adverse reactions.

The core advantage of CP91110P aligns with the aforementioned characteristics of epitope vaccines, and its antigen selection strategy—covering both the active and latent phases of MTB infection—directly addresses the key drawbacks of traditional vaccines. Unlike BCG, which only targets early infection and cannot prevent the reactivation of latent TB, CP91110P integrates epitopes from 3 proliferative-phase antigens (Ag85A, Ag85B, Rv0288) and 2 latent-phase antigens (Rv1813c, Rv2031c). Ag85A and Ag85B, major secreted MTB antigens, are involved in cell wall synthesis and adhesion, and their inclusion aligns with their proven immunogenicity in vaccines such as H56:IC31 and MVA85A [5,105]. Rv0288 (an ESAT-6 family protein) is recognized by T cells in TB patients and BCG-vaccinated individuals, enhancing responses against active infection [27,106]. For latent infection, Rv1813c modulates host cell metabolism and is recognized by over 70% of TB patients [107,108], while Rv2031c (regulated by the DosR regulon) maintains MTB dormancy under hypoxia, making it a critical target for latent phase control [109]. Compared with other MEVs (Table S4), CP91110P achieves more balanced coverage of infection stages. For example, W541 includes proliferative and latent antigens but lacks ESAT-6 family proteins like Rv0288, which are key for early immune recognition [110]. ZL9810L [6] and ZL12138L [15] focus on Ag85A/B and latent antigens (Mtb39A, Rv1980c, Rv3873) but omit Rv1813c, a highly immunogenic modulator of host–pathogen interactions. Meanwhile, vaccines such as PP13138R include a broader range of antigens but lack the targeted selection of immunodominant epitopes from both phases, potentially diluting the immune focus [21]. Thus, CP91110P’s antigen selection strikes a balance between breadth (covering active/latent stages) and specificity (prioritizing immunodominant epitopes), enhancing its potential to combat both infection phases.

CP91110P is predicted to exhibit strong immunogenicity and antigenicity, supported by both rational design strategies and computational validation. From a design perspective, the vaccine prioritizes immunodominant epitopes filtered for non-toxicity and non-allergenicity, ensuring efficient recognition by the immune system. Additionally, the integration of TLR-2 agonist PorB, TLR-4 agonist HBHA, and PADRE peptide synergistically enhances immune activation: PorB triggers innate immunity via TLR-1/TLR-2 complexes [45,46,111,112,113], HBHA promotes Th1 differentiation and CTL responses [114], and PADRE broadens MHC-II binding [115], collectively bridging innate and adaptive immunity. Computational analyses further validate these properties. CP91110P’s antigenicity score (VaxiJen: 0.8789, threshold > 0.5) is comparable to or higher than many reported MEVs, such as W541 (0.6527) and 50 sRP-TB (0.6375–0.873047) [110,116], and its immunogenicity score (IEDB: 4.40091) exceeds ZL9810L (2.21451) [6] and ZL12138L (4.14449) [15] (Table S4). Structural features, including a high proportion of random coils (60.46%)—which facilitate TLR-binding site exposure—and stable tertiary structure (98.34% in Ramachandran-favored regions, MolProbity score has been increased to 0.66), further support efficient immune recognition. Immune simulations predict robust responses, including NK cell activation, macrophage infiltration (peaking at 3–5 days post-vaccination), and Th1 polarization (elevated IFN-γ/IL-2, suppressed IL-4), which are critical for clearing intracellular MTB. Notably, CP91110P’s strong binding to TLR-4 (−1672.5 kcal/mol) and TLR-2 (−1535.9 kcal/mol) indicates its immunogenicity. However, the high affinity for TLR-4 raises concerns about potential excessive inflammation, as TLR-4 overactivation is linked to pathological responses (e.g., cytokine storms) [117,118]. Nevertheless, molecular dynamics simulations revealed flexible interactions (RMSD: 2–6 Å for TLR-4 complex), which may mitigate persistent over-signaling, suggesting a potential intrinsic regulatory mechanism.

CP91110P is predicted to exhibit favorable physicochemical properties that support its potential for clinical translation, outperforming many reported MEVs in key parameters (Table S1). Its instability index (33.48) is lower than that of W541 (45.37), indicating better structural stability [110], while its solubility (0.485) exceeds the threshold (0.45) for aqueous formulation, comparable to ZL12138L (0.47) [15] and superior to vaccines with unreported solubility (e.g., W541) [110]. The predicted half-life in mammalian reticulocytes (20 h) is sufficient for sustained immune stimulation, exceeding the 1.1 h of W541 and matching the 20–30 h of advanced candidates like ZL12138L and PP19128R [15,21,110]. In terms of production feasibility, CP91110P shows high expression potential in *E. coli* (CAI: 0.746) and includes an N-terminal His-tag for straightforward purification, addressing scalability challenges in resource-limited settings. This contrasts with vaccines requiring complex expression systems or exhibiting low solubility, which hinder large-scale production. Collectively, these properties position CP91110P as a translationally viable candidate, balancing stability, solubility, and manufacturability.

CP91110P is predicted to achieve a global HLA-I/II coverage of 86.18%, which is competitive with other MEVs (Table S1). For instance, it exceeds PP13138R (52.42–79.69%) and ZL9810L (72.89–81.49%) and is comparable to ZL12138L (92.41–90.17%) and PP19128R (82.24–93.71%), highlighting its broad applicability [15,20,21]. However, significant regional disparities were observed: coverage is notably lower in South Africa (37.00%) and Southeast Asia (68.83%) compared to the global average. This variation is driven by population-specific HLA allele distributions (Table S5). In South Africa, key vaccine-targeted alleles (e.g., HLA-DRB1*07:01, HLA-A*03:01 and HLA-A*68:02) are either absent or present at very low frequencies (<0.1) in local populations (e.g., South African Black, Limpopo Venda and Caucasians). In Southeast Asia, exemplified by Malaysian and Vietnamese cohorts, HLA molecules corresponding to vaccine epitopes (e.g., HLA-DRB1*07:01, HLA-B*35:01) are underrepresented, impairing effective epitope presentation. These findings emphasize the need to integrate geographically specific HLA profiles into MEV design to enhance regional coverage.

Despite promising in silico results, CP91110P has several limitations. First, all findings rely on computational predictions. While molecular docking and MD simulations indicate stable interactions with TLR-2 and TLR-4, functional engagement of these receptors depends not only on binding affinity but also on receptor conformational changes, co-receptor involvement, and downstream signaling—factors that cannot be reliably inferred from static docking scores or dynamic simulation data alone. Thus, wet-lab studies are needed to confirm epitope immunogenicity (via ELISPOT/IFN-γ assays) and validate TLR-2/4 signaling activation (e.g., through reporter assays for NF-κB kinetics in PBMCs or detection of downstream cytokine production). Second, regional HLA coverage deficits in South Africa and Southeast Asia necessitate optimization, either by adding epitopes targeting high-frequency alleles in these regions or designing region-specific vaccine variants. Notably, HLA coverage predicted in silico may not directly translate to actual vaccine effectiveness across diverse populations. Computational models rely on known HLA-epitope binding affinities and population allele frequencies, but real-world efficacy is influenced by additional factors: epitope processing efficiency, competition between epitopes for MHC binding, and host genetic backgrounds affecting immune cell function [119,120,121]. These discrepancies highlight the need for post-vaccination monitoring in diverse cohorts to validate computational predictions. Third, the potential for excessive inflammation due to strong TLR-4 binding requires mitigation strategies, such as epitope modification to fine-tune affinity, adjuvant formulation adjustments, or integration of anti-inflammatory motifs (e.g., IL-10-inducing sequences). Fourth, MTB strains may evade immune recognition through epitope mutation or downregulation of antigen expression, which could potentially compromise the long-term efficacy of CP91110P given its reliance on specific immunodominant epitopes identified in this study. Future studies should therefore evaluate the vaccine’s cross-reactivity against clinically prevalent MTB variants to address such immune evasion risks. Finally, the scalability of the pET28a(+) expression system in industrial bioreactors needs assessment to ensure accessibility in resource-limited settings. Addressing these limitations through a pipeline of computational refinement and experimental validation will be critical to translating CP91110P into a safe, effective, and globally applicable TB vaccine.

## 5. Conclusions

In conclusion, the innovative design of CP91110P holds potential for advancing TB vaccination strategies by targeting both latent and active infections. In the computer simulation predictions, the integration of the immunodominant epitopes and the adjuvant’s synergistic effect indicates that this is a reliable platform capable of triggering a strong immune response. However, addressing the highlighted limitations through further empirical studies and optimization will be crucial in translating these findings into effective public health interventions. Maybe by overcoming these challenges, CP91110P could significantly contribute to controlling TB, particularly in LMICs, ultimately reducing the disease burden on vulnerable populations.

## Figures and Tables

**Table 1:** The HTL, CTL, and B-cell epitopes selected from five antigens to construct the CP91110P vaccine.

Protein	Peptide Sequence	Length	Alleles	Percentile Rank ^a^	Antigenicity Score ^b^	IFN-γ Score ^c^	Immunogenicity Score ^d^	ABCpred Score ^e^	Allergen FPv.2.0 ^f^	Toxin Pred ^f^	IL-4 ^g^	IL-10 ^h^
**HTL epitopes**												
Ag85A	VGAVGGTATAGA	12	HLA-DQA1*05:01/DQB1*03:01	0.27	1.588	0.56383711	N/A	N/A	Non	Non	Non	Non
	PVEYLQVPSPSMGRD	15	HLA-DRB1*07:01	0.32	0.7094	0.99952242	N/A	N/A	Non	Non	Non	Non
	LPVEYLQVPSPSMGRD	16	HLA-DRB1*07:01	0.43	0.9243	0.7434204	N/A	N/A	Non	Non	Non	Non
Ag85B	VGLAGGAATAGA	12	HLA-DQA1*05:01/DQB1*03:01	0.06	1.2823	0.70650331	N/A	N/A	Non	Non	Non	Non
	LPVEYLQVPSPSMGRD	16	HLA-DRB1*07:01	0.43	0.9243	0.7434204	N/A	N/A	Non	Non	Non	Non
	SANRAVKPTGSAAIGLSM	18	HLA-DRB1*07:01	0.21	0.7624	0.94857343	N/A	N/A	Non	Non	Non	Non
	PVEYLQVPSPSMGRD	15	HLA-DRB1*07:01	0.32	0.7094	0.99952242	N/A	N/A	Non	Non	Non	Non
	GLAGGAATAGAF	12	HLA-DQA1*05:01/DQB1*03:01	0.33	0.9538	0.62913663	N/A	N/A	Non	Non	Non	Non
Rv0288	VRAYHAMSSTHEANTMA	17	HLA-DRB1*04:05	0.12	0.8572	0.76415085	N/A	N/A	Non	Non	Non	Non
Rv1813c	YNGSKYQGGTGLTRRAA	17	HLA-DRB5*01:01	0.42	1.2302	0.14674751	N/A	N/A	Non	Non	Non	Non
	NLRRRTAMAAAGLGAALG	18	HLA-DPA1*02:01/DPB1*14:01	0.48	0.6188	0.92377101	N/A	N/A	Non	Non	Non	Non
**CTL epitopes**												
Ag85A	RVWVYCGNGK	10	HLA-A*03:01	0.42	0.7151	N/A	0.10358	N/A	Non	Non	N/A	N/A
	MGPTLIGLAM	10	HLA-B*35:01	0.23	0.524	N/A	0.19966	N/A	Non	Non	N/A	N/A
		10	HLA-B*07:02	0.47								
Ag85B	NAAGGHNAV	9	HLA-A*68:02	0.2	1.9957	N/A	0.12765	N/A	Non	Non	N/A	N/A
	NAAGGHNAVF	10	HLA-B*35:01	0.33	1.4758	N/A	0.16235	N/A	Non	Non	N/A	N/A
	GLAGGAATA	9	HLA-A*02:03	0.13	1.3338	N/A	0.17233	N/A	Non	Non	N/A	N/A
Rv0288	STHEANTMA	9	HLA-A*68:02	0.34	0.9084	N/A	0.07342	N/A	Non	Non	N/A	N/A
Rv1813c	KYQGGTGLTR	10	HLA-A*31:01	0.24	1.3245	N/A	0.11252	N/A	Non	Non	N/A	N/A
Rv2031c	GLRPTFDTR	9	HLA-A*31:01	0.14	1.7146	N/A	0.19504	N/A	Non	Non	N/A	N/A
	RPTFDTRLM	9	HLA-B*07:02	0.09	1.3402	N/A	0.22574	N/A	Non	Non	N/A	N/A
		9	HLA-B*35:01	0.41								
**B cellular epitopes**												
Ag85A	DFSGWDINTPAFEWYD	16	N/A	N/A	N/A	N/A	N/A	0.9	Non	Non	N/A	N/A
	SDMWGPKEDPAWQRND	16	N/A	N/A	N/A	N/A	N/A	0.9	Non	Non	N/A	N/A
Ag85B	ADMWGPSSDPAWERND	16	N/A	N/A	N/A	N/A	N/A	0.9	Non	Non	N/A	N/A
	AGGYKAADMWGPSSDP	16	N/A	N/A	N/A	N/A	N/A	0.88	Non	Non	N/A	N/A
	LRAQDDYNGWDINTPA	16	N/A	N/A	N/A	N/A	N/A	0.85	Non	Non	N/A	N/A
	GGAATAGAFSRPGLPV	16	N/A	N/A	N/A	N/A	N/A	0.85	Non	Non	N/A	N/A
Rv0288	PAMLGHAGDMAGYAGT	16	N/A	N/A	N/A	N/A	N/A	0.85	Non	Non	N/A	N/A
Rv1813c	YGAIAYAPSGASGKAW	16	N/A	N/A	N/A	N/A	N/A	0.92	Non	Non	N/A	N/A
	EDDAVNRLEGGRIVNW	16	N/A	N/A	N/A	N/A	N/A	0.85	Non	Non	N/A	N/A
Rv2031c	VRAELPGVDPDKDVDI	16	N/A	N/A	N/A	N/A	N/A	0.88	Non	Non	N/A	N/A

^a^ Percentile rank: Epitopes with a percentile rank < 0.5 were prioritized. ^b^ Antigenicity score: Epitopes scoring > 0.5 were selected. ^c^ IFN-γ induction: Epitopes with positive scores and the highest predicted IFN-γ secretion capacity were retained. ^d^ Immunogenicity score: Epitopes were ranked and selected based on descending immunogenicity scores. ^e^ Linear B-cell epitope score: Epitopes were prioritized according to descending prediction scores. ^f^ Allergenicity/toxicity: Epitopes labeled “Non” (non-allergenic and non-toxic) were chosen. ^g^ IL-4 suppression: Epitopes lacking predicted IL-4 induction were selected. ^h^ IL-10 suppression: Epitopes without predicted IL-10 induction were retained. N/A, not applicable.

**Table 2:** Global population coverage of the CP91110P vaccine for combined HLA I and II alleles.

Population/Area	Class Combined		
	Coverage ^a^	Average_Hit ^b^	pc90 ^c^
Central Africa	81.63%	3.43	0.54
Central America	87.71%	4.04	0.81
East Africa	83.67%	3.55	0.61
East Asia	75.12%	2.81	0.4
Europe	87.68%	4.55	0.81
North Africa	78.89%	4.02	0.47
North America	90.28%	5.1	1.09
Northeast Asia	78.68%	3.23	0.47
Oceania	79.98%	3.28	0.5
South Africa	37.00%	0.8	0.16
South America	91.34%	4.49	1.15
South Asia	83.97%	3.98	0.62
Southeast Asia	68.83%	2.58	0.32
Southwest Asia	75.59%	3.38	0.41
West Africa	97.85%	5.86	3.02
West Indies	79.97%	3.9	0.5
World	86.18%	4.17	0.72
Average	80.26	3.72	0.74
Standard deviation	12.47	1.07	0.62

^a^ Projected population coverage. ^b^ Average number of epitope hits/HLA combinations recognized by the population. ^c^ Minimum number of epitope hits/HLA combinations recognized by 90% of the population.

**Table 3:** The predicted parameters of the CP91110P vaccine.

Parameters		Results
Biological characteristics	Antigenicity	0.8789 ^a^
		0.801088 ^b^
	Immunogenicity	4.40091
	Sensitization	Non
	Toxicity	Non
Physicochemical properties	Number of amino acids	784
	Molecular weight(Da)	80,722.29
	Theoretical pI	7.38
Estimated half-life (h) ^c^	Mammalian reticulocytes (in vitro)	20
	Yeast (in vivo)	0.5
	*E. coli* (in vivo)	>10
	Instability index	33.48
	Aliphatic index	66.07
	Grand average of hydropathicity (GRAVY)	33.48
Basic features	Solubility	0.485

^a^ The score of antigenicity predicted by the VaxiJen v2.0 server. ^b^ The score of antigenicity predicted by the ANTIENpro server. ^c^ The half-life of a candidate sequence in hours.

**Table 4:** MM-PBSA Binding Free Energy Components of CP91110P–TLR Complexes.

Component	CP91110P–TLR2 (kcal/mol)	CP91110P–TLR4 (kcal/mol)
ΔEvdw (van der Waals)	−40.8	−35.6
ΔEele (Electrostatic)	−120.3	−105.7
ΔGPB (Polar solvation)	110.8	102.4
ΔGSA (Non-polar solvation)	−9.2	−7.8
ΔGbind Total	−59.5	−46.7

**Table 5:** MM-GBSA Binding Free Energy Components of CP91110P–TLR Complexes.

Component	CP91110P–TLR2 (kcal/mol)	CP91110P–TLR4 (kcal/mol)
ΔEvdw (van der Waals)	−40.8	−35.6
ΔEele (Electrostatic)	−120.3	−105.7
ΔGGB (Polar solvation)	102.5	95.3
ΔGSA (Non-polar solvation)	−9.2	−7.8
ΔGbind Total	−67.8	−53.8

## Data Availability

Data are contained within the article and Supplementary Materials.

## Supplementary Materials

The following supporting information can be downloaded at: https://www.mdpi.com/article/10.3390/biology14091196/s1. Table S1: Among the 5 MTB antigens of the CP91110P vaccine, HTL and CTL epitopes with percentile rank < 0.5 were selected; Table S2: Conformational B-Cell Epitopes in CP91110P vaccine; Table S3: Potential Disulfide Bonds in CP91110P: Residue Pairs, χ3 Torsion Angles, Bond Energies, and Σ B-Factors; Table S4: Comparative Analysis of Vaccine Candidates Based on Antigen Characteristics and Predicted Properties; Table S5: HLA alleles with a frequency higher than 1% in populations from South Africa and Southeast Asia (Hong Kong, Taiwan, Thailand, Singapore, Malaysia and Vietnam).
